# The Characteristics of the ‘Good Nurse': A Scoping Literature Review

**DOI:** 10.1155/nrp/8460996

**Published:** 2025-08-05

**Authors:** Ali Haddad

**Affiliations:** Nursing Department, Amiri Hospital, Sharq, Kuwait

**Keywords:** character, competence, ethical characteristics, good nurse, ICU

## Abstract

**Aim:** To explore nurses' and patients' perspectives on characteristics of the ‘good nurse' (an ethical ideal, representing perfection in nursing practice) in hospitals.

**Design:** Scoping literature review and narrative synthesis.

**Methods:** The scoping literature review was undertaken using the Preferred Reporting Items for Systematic Reviews and Meta-Analyses (PRISMA) methodology. Subsequently, a thematic analysis of identified papers was undertaken to uncover the main themes relating to characteristics of the good nurse.

**Data Sources:** Five databases (Medline, Cumulative Index for Nursing and Allied Health Professionals (CINAHL), British Nursing Index, Scopus and PsycINFO) were searched in May 2023. An initial 2968 records were identified, which were refined to 124 articles after screening titles and abstracts, and then further refined through more detailed review to 13 articles meeting the inclusion criteria for the study.

**Results:** Two central themes were identified: character and competence. With respect to the good nurse's character, the virtues which were identified as important include patience, being caring, optimism, trustworthiness, selflessness, respectfulness and confidence. With respect to competence, the studies revealed that the good nurse must demonstrate knowledge and technical skill, act in a professional way, be an expert communicator, be supportive to patients and colleagues and know how to provide holistic care.

**Conclusions:** The results highlight the dual importance of character and competence in defining the good nurse. Nurses and patients both recognised the importance of balancing technical expertise with character traits and effective communication to offer the best possible care.

**Summary**

• Implications
◦ The results provide valuable insights for nurse practitioners, managers and educators.
◦ In particular, they suggest that training programmes should focus on developing interpersonal qualities and character alongside technical expertise to improve patient care.
• Reporting method
◦ The review adheres to PRISMA guidelines for the reporting of systematic reviews.
• Patient or public contribution
◦ No patient or public contribution: Scoping review

## 1. Introduction

Existing frameworks for nursing expertise have determined how standards of excellence in the profession are shaped and assessed. For example, Benner's novice-to-expert model [1] conceptualises five stages of clinical competence, concluding with the expert nurse whose vast experience and clinical skills enable them to act efficiently, flexibly and with deep contextual understanding. Similarly, the International Council of Nurses' (ICN) Framework of Competencies for the Nurse Specialist [2] emphasises the need for high-quality education to develop expert clinical judgement and skills such as leadership and mentoring, research and evidence-based practice, care management, health promotion and patient advocacy. However, these frameworks place comparatively less emphasis on the moral, relational and character-based dimensions of nursing, creating a conceptual gap between what is measured and what is often most valued in practice.

A more holistic representation of nursing excellence is provided through the construct of the ‘good nurse', referring to an ethical ideal which emerged alongside the professionalisation of nursing across the 20^th^ century. As Fealy [3] describes, nurses originally had the social status of a domestic servant, as they were typically untrained and recruited from the poorer classes. However, perceptions started to change following widespread public recognition of nurses' extraordinary efforts during the First World War. Hereafter, nurses began to enjoy a position of high public esteem. Commentators began to laud nurses' ‘devotion to duty, self-sacrifice, heroism and a willingness to serve the medical profession' (p. 651), extolling the personal attributes, dispositions and qualities that were held to be particularly important in the performance of the nursing role. Eventually, this resulted in the ‘good nurse' ideal; an archetypal representation of nursing perfection captured in the image of an angelic and ever-present provider of flawless care, whose character virtues enable expert navigation of challenges and sustained excellence in nursing [4].

In this way, the good nurse is an image of perfection which is impossible to live up to in reality, and which may even be harmful in the sense that it creates unrealistic expectations for nurses [4–6]. Nonetheless, it is a useful construct from the research perspective, because it helps to reveal the personal qualities and skills which are thought to be most conducive to expert nursing. As such, we define the ‘good nurse' as both an aspirational ethical ideal (representing the highest standards of moral and professional conduct) and as a functional concept that can guide education and training. While the idealised version may be unattainable in its entirety, it serves as a benchmark that highlights the character traits and competencies most valued in effective nursing practice, informing frameworks for nursing education and training.

In this review, we aim to explore both interpretations: the good nurse as an unreachable archetype and as a realistic model shaped by nurses' and patients' perceptions of excellence in hospital care. This follows the work of numerous researchers in recent decades, who have sought to identify the skills and characteristics of the good nurse. In particular, three previous review papers have summarised the literature on the good nurse.

Van der Elst et al. [7] explored perceptions of the good nurse amongst elderly patients and residents in particular. In their results, they distinguished two themes: characteristics of the good nurse when it comes to physical care (e.g., technical expertise, availability, ensuring patients' comfort and attention to detail) and characteristics of the good nurse when it comes to psychosocial care (e.g., friendliness, patience, honesty, sincerity, responsibility, compassion and responsiveness).

Another review was conducted by Rchaidia and colleagues [8], who explored cancer patients' perceptions of the good nurse. They found that the characteristics of the good nurse could be grouped into two categories: attitude (e.g., friendliness, sincerity, respectfulness, sensitivity, optimism, honesty, relating to the patient as a person) and knowledge and skills (e.g., professional experiential knowledge, technical competence, able to provide detailed advice).

Finally, Lee and Kim [9] conducted an integrative review of the good nurse from the perspective of both patients and nurses. Synthesising the results of 11 studies, they found that the good nurse could be defined in terms of personality traits (e.g., regardful, humanistic, supportive, faithful) and professional competence (e.g., professional manner, practical quality, patient-centred care, communication).

Overall, the three reviews highlighted very similar qualities of the good nurse. Moreover, each of the reviews delineated the personality and attitudinal qualities of the good nurse from those which were associated with professional knowledge and competence. In the present study, we aim to build upon the review of Lee and Kim [9] in particular, with three key differences. Firstly, their review did not examine the ethical or cultural underpinnings of good nursing traits, which our study addresses through integration with virtue ethics and cultural models. Secondly, their intention to explore differences in the perceptions of nurses and patients was thwarted by only identifying two studies conducted with nurses, which we aim to overcome through an even more comprehensive search of the academic literature. Thirdly, three of the 11 studies they identified were conducted with child samples, whereas we intentionally exclude children from our study, as their perceptions may be unduly influenced by stereotypes rather than by their own observations.

Overall, therefore, a scoping review was used to survey previous research on the characteristics of the good nurse. The review sought to answer the research question: What are nurses' and patients' perspectives of the good nurse in hospital settings? Hospital settings present a unique environment where the demands on nursing staff are particularly intense, making it an ideal context for examining the characteristics required for excellence in nursing care.

## 2. Methods

### 2.1. Study Design

A scoping review was chosen as the literature review method. This method makes it possible to synthesise existing literature to identify its scope and value, and to identify research gaps, without necessarily conducting a full systematic review [10]. It was chosen for this review as it is recognised to be the most appropriate form of literature review when the intention is to identify, map, report and discuss characteristics or concepts from the research literature [11].

The suggested framework for scoping reviews within the Joanna Briggs Institute (JBI) scoping review manual guide [12] was followed, entailing the identification of the research question, identification of relevant studies, study selection, charting the data and summarising and reporting the results. The optional stakeholder consultation stage was not completed, as the aim of the review was to synthesise existing peer-reviewed evidence rather than to inform the development of specific interventions or policies.

The scoping review was conducted by a single author, as part of his PhD research. Specifically, the author was responsible for performing the database searches, eligibility screening, data charting, study appraisal and thematic coding. The review was closely supervised by three academic advisors with expertise in both clinical nursing and scoping review methodology. A university librarian also reviewed and approved the search strategy to ensure its comprehensiveness and alignment with best practices. The academic supervisors guided and cross-checked each stage of the review, including the search strategy, inclusion decisions and development of the coding framework. Disagreements about study selection, appraisal or coding decisions were resolved through discussion during regular supervisory meetings. Although the review was formally conducted by one person, the ongoing oversight and iterative feedback process helped to ensure its rigour and validity.

### 2.2. Eligibility Criteria

To be eligible for inclusion in the literature review, the paper must be a primary research study from any year, written in English and published in a peer-reviewed academic journal. There were no restrictions based on publication date, so that all relevant papers could be retrieved. Grey literature (e.g., theses, dissertations and conference proceedings) was excluded to ensure consistency in methodological quality and reporting standards across included studies.

Studies were eligible if they focused on perceptions of the good nurse in hospital settings, from the perspective of either nurses or adult patients. Studies were excluded if they reported secondary research. Studies were also excluded if they reported the perspectives of children, due to the risk that their perception of the good nurse would be influenced by stereotypes and media portrayals. Finally, studies were also excluded if they considered nursing in any other context than hospitals, as the meaning of being a good nurse might substantially differ in these settings.

### 2.3. Search Strategy

A systematic search process was followed to identify, appraise and synthesise all relevant literature pertaining to the research topic. Articles were identified in the following five databases: Medline, Cumulative Index for Nursing and Allied Health Professionals (CINAHL), British Nursing Index, Scopus and PsycINFO. Example search results from Medline and Scopus are provided in the Supporting Information (available here). These databases were selected because they encompassed the fields which are most relevant for this study, including nursing, medicine, social sciences, philosophy and ethics.

Search terms and keywords were developed using the JBI guide [12], which recommends that a search is conducted within three categories: population, concept and context. This approach is particularly appropriate for reviews that deal with broader, less restrictive inclusion criteria [12]. Applied to this review, the population comprises nurses and patients who have reported their perceptions, concept is the perception of the good nurse, and context is the hospital setting. From this, the more specific search criteria can be developed, shown in Table 1.

### 2.4. Search Process

**Figure 1:**
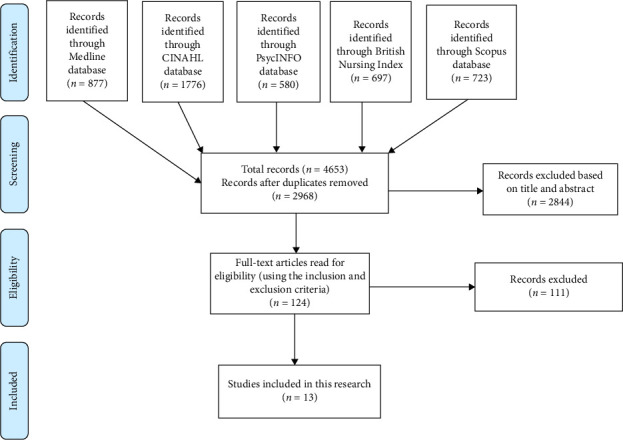
Diagram of the PRISMA process for studies into the characteristics of the good nurse.

The Preferred Reporting Items for Systematic Reviews and Meta-Analyses (PRISMA) diagram (Figure 1) shows the search process. An initial search across five databases identified 4653 records, which were imported into EndNote X8 for reference management and deduplication. After removing 1685 duplicates, 2968 records remained for screening. The lead author reviewed the titles and abstracts of the identified studies to judge whether they potentially met the eligibility criteria. Based on this assessment, 2844 records were excluded for being clearly out of scope. The remaining 124 articles were retrieved and read in full to allow for a more detailed judgement. Of these, 111 were excluded at the full-text stage, leaving 13 studies that met the inclusion criteria in full.

### 2.5. Data Charting

A data charting process was followed to present a descriptive summary of the identified literature as it related to the review question. Specifically, Table 2 at the end of this section shows the different aspects that were charted for each study, including the study author(s), the year of publication, the aims and objectives, the country in which study took place, the study population and sample size, the research and analytic method and the key findings that relate to the review questions.

Table 2 also summarises the results of a critical appraisal process, using the Critical Appraisal Skills Programme (CASP) checklist [13, 26] (for the 12 qualitative or mixed-methods studies) or the Critical Appraisal of a Cross-Sectional Study (Survey) tool [14] (for the quantitative study) to assess the quality of each study. These tools do not have their own scoring systems, so a pragmatic rule-based system was developed whereby a study was judged as high quality if it met 100% of the quality criteria, as moderate quality if it met 60%–99% of the quality criteria or low quality if it met less than 60% of the quality criteria. Each of the 13 studies identified in the review [15–25, 27, 28] was rated as either moderate or high quality. Because all the studies had acceptable quality, the quality ratings were not used to inform the weighting of themes during synthesis, but rather included as evidence that all studies met the basic threshold for methodological soundness.

The data charting process was developed by the author in line with review objectives. While it was not pilot tested or subjected to a formal calibration exercise, the extracted data were regularly reviewed and discussed with academic supervisors to ensure accuracy and consistency.

### 2.6. Data Analysis

Finally, a six-phase thematic analysis process was used to construct themes and subthemes relating to the characteristics of the good nurse [29]. First, each article was read and re-read numerous times so that we became acquainted with the data. Next, initial codes were generated, identifying the features of the data which were particularly interesting and meaningful (i.e., the reported character traits and behaviours of the good nurse). Coding was conducted using NVivo, which supported the organisation and retrieval of coded data. The list of initial codes was then grouped into overarching themes and subthemes by identifying patterns across studies and related concepts. This early coding framework was then developed and refined iteratively throughout the review, with regular discussions and feedback from supervisors to review theme boundaries, check interpretive consistency and resolve any uncertainties. I maintained reflective notes throughout the process to track analytic decisions and examine how my own assumptions might have shaped interpretation. After my supervisors and I had reached consensus regarding the framework, the final themes and subthemes were defined and named, using names and definitions that captured the essence of the concept in a concise but accurate manner. Finally, a written report of the findings was produced (summarised in the following section).

## 3. Results

Of the 13 included studies, six focused on the perspectives of nurses, six on the perspectives of patients, and one included both nurses and patients. The results of the thematic analysis are shown in Table 3. It shows the two overarching themes defining the good nurse (character and competence) and their constituent subthemes, as well as the related terms identified in the studies.

### 3.1. Character

Character describes the individual's moral qualities and inclinations, reflecting the presence or absence of virtues [30]. Thus, it reflects the traits that underpin moral and intellectual excellence in nursing. The nurse cultivates, refines, and habituates these traits, leading to a disposition to act in a ‘good' way (supporting the well-being of their patients, particularly in times of difficulty and/or moral uncertainty) [15, 19].

#### 3.1.1. Patience

Patience (the capacity to accept or tolerate delay, problems or suffering without becoming annoyed or anxious) was highlighted as an important character trait for the good nurse in six out of 13 studies, including the perspectives of both patients and nurses [17–19, 21, 22, 28]. In one mixed-methods study [22], patience was identified as the top-ranked good nursing trait out of 33 possible options. In another interview study [28], patients identified being a ‘tower of patience' as a core characteristic of the perfect nurse, believing that it would help the nurses to more effectively deal with the inconveniences and hardships they encountered, and also help when reassuring stressed patients. Other studies highlighted the related qualities of calmness (the ability to maintain a clear mental focus and avoid annoyance, anxiety or other strong emotions) [17, 20, 22] and the requirement to be open and flexible to change [19]. Overall, therefore, the studies show that both patients and nurses identified an expectation for the good nurse to be patient.

#### 3.1.2. Caring

There was widespread agreement that the good nurse should be caring (i.e., possess an attitude inclined towards concern for the well-being of others). This was explicitly highlighted in nine out of the 13 studies [15–22, 28] and implicitly underscored in the other four studies through discussions of how nurses demonstrate caring behaviours [23–25, 27]. It was particularly important in the study by Catlett and Lovan [19], where it was highlighted as one of the four main categories of the good nurse. Summarising its importance, one interviewee in this study said, ‘(the good nurse) has to be someone who really cares about other people. They are a giver and have that personality that they are a caregiver and like to care for other people' (p. 59). Other studies highlighted the related traits of compassion (i.e., the ability to recognise patients' hardships and strive to provide comfort) [15, 16, 19, 21, 25, 27, 28], as well as being attentive, considerate and diligent [15, 19, 20, 22, 23].

Certain terms were only highlighted in studies with patients. For example, patients in three studies [16, 17, 28] highlighted that good nurses should be sympathetic. Interestingly, these studies had not identified empathy as an important requirement. This possibly reflects a belief that it is not really possible for the nurse to share the patient's experience without directly experiencing it themselves, but nonetheless, it is appreciated when the nurse understands and acknowledges the patient's struggles. Other good nursing characteristics only identified by patients included being sensitive [16, 18] and gentle [16], suggesting that it is the recipients of care who are most mindful of the importance of interpersonal qualities.

#### 3.1.3. Optimism

Numerous studies indirectly referred to the need for the good nurse to be optimistic (i.e., possessing the tendency to see the world in a hopeful and positive light, even in the face of adversity). For example, two studies with nurses in South Korea both highlighted positivity as one of the main categories describing the good nurse [24, 25], relating the concept to self-efficacy. Other studies confirmed that both nurses and patients understood the importance of optimism, highlighting the traits of being friendly [18, 22], warm [21, 24], kind [18, 19] and enthusiastic [22].

Studies with patients provided further detail as to why such traits were valued. The importance of being cheerful, smiley, kind and warm was highlighted in a study with cancer patients [16], with one patient saying that ‘A patient (like me) feels comfortable and easy to say something to a nurse like that' (p. 19). A study of mental health patients highlighted the importance of encouragement [21], perhaps indicating that those recovering from mental health issues are particularly appreciative of nurses who are optimistic.

Interestingly, most studies highlighting optimism were in Asian or Middle Eastern countries (with only one exception [18], which was in the UK), suggesting that the trait is particularly valued in these cultures. There are likely also cultural differences within Asian and Middle Eastern cultures. For example, even though the study in Japan [16] identified the importance of nurses being cheerful and warm, they also highlighted that becoming overly familiar with patients would be seen as disrespectful and unprofessional. The authors attributed this to the fact that the ‘Japanese have a strong sense of inside and outside the family' (p. 18), so the good nurse in Japan needed to maintain a degree of separation with their patients in order to act in a culturally appropriate manner. These cultural differences suggest that training programmes may need to be tailored to emphasise optimism more in certain regions, whereas in other regions, maintaining professional distance might be more appropriate.

#### 3.1.4. Trustworthiness

Several traits were highlighted which together reflected the perception that the good nurse must be trustworthy. For example, honesty was highlighted as an important requirement in two studies of patients [17, 18] and one study of nurses [19]. However, each of these simply included honesty in a list of important traits without further elaboration, so the specific reasons for its importance could not be ascertained. Possibly, the lack of elaboration may reflect a perception that is an inherent expectation of nursing roles.

Other studies highlighted related traits, describing how the good nurse should be reliable [16], dependable [19], loyal [18, 19], nonjudgemental [19], approachable [17] and sincere [22]. All of these traits describe ways in which the nurse can build further trust in their relationships with patients. Finally, one study identified the requirement for the good nurse to be ethical [19] (having the capacity for ethical decision-making and ‘doing the right thing'), which is also related to trustworthiness in the sense that it inspires confidence that the nurse will act in an appropriate and caring way.

#### 3.1.5. Selflessness

Selflessness (i.e., showing greater concern for patients' needs and wishes than their own) was highlighted in three studies [15, 19, 20], albeit to a varying extent in each. Only one [19] actually used the term selfless, including it within a list of traits that the good nurse should have, but without elaborating on why it was important. It was described more indirectly in other studies. For example, one discussed the theme of patient-centredness [15] (i.e., the tendency to prioritise the patient's individual needs, preferences and values in all aspects of care) and referred to nurses' descriptions of the good nurse as one who ‘always keeps patient needs and desires first' and ‘puts her patient's best interests first' (p. 306). Just one study with patients identified the requirement for selflessness [20], through the dimension of ‘(putting the patient) first no matter what else happens'. This was a constituent item of a broader trait identified as an important characteristic of the good nurse; however, ratings for specific items were not provided, so its specific importance could not be ascertained. The relatively limited discussion of selflessness within the identified studies could reflect a broader cultural shift towards recognising the importance of nurse self-care, though it remains a crucial attribute of patient-centred care.

#### 3.1.6. Respectfulness

Respectfulness (i.e., showing courteous regard for other people's feelings) was identified in six of the 13 studies [15–18, 20, 21]. Only one [15] was conducted with nurses, showing that patients had a greater appreciation of respectfulness than the nurses did themselves, possibly due to their experiences of being treated with a lack of respect. Indeed, a perceived lack of respect was identified in an interview study of adults with disabilities and their carers [17], with interviewees recommending that nurses ‘recognise that we are grown-ups' and ‘remember they (patients with disabilities) are just people too' (p. 383). Similarly, respectfulness also emerged as an important theme amongst mental health patients [21], who expressed a desire to be treated as whole persons and not merely as cases of their illness. The patients in both studies were in vulnerable conditions, which may explain why they particularly valued respectfulness. One additional study with nurses [25] did indirectly acknowledge a similar viewpoint on the importance of respectfulness, through a description of the need to ‘preserve personhood' in patients.

#### 3.1.7. Confidence

Finally, confidence (i.e., belief in one's own abilities, qualities and judgements) was directly highlighted in one study with patients [17], where it was included in a list of core attributes of the good nurse, but with no further elaboration to describe why it was important. Other studies used similar descriptors to allude to the importance of confidence. For example, a study of both patients and nurses [25] identified self-efficacy as one of the five main factors describing the good nurse, as belief in one's own ability was judged to help nurses to confront and manage diverse challenges. Other studies found that the good nurse must be courageous [18, 19] and firm [17] in their decision-making. Overall, although confidence was only mentioned directly in one study, its indirect references through related traits suggest that it plays a foundational role in enabling nurses to perform competently under pressure.

### 3.2. Competence

The second core component of the good nurse, alongside a virtuous character, was competence (i.e., the necessary ability, knowledge and skill to effectively provide high-quality care to patients). This reflects the nurses' knowledge and expertise as it pertains to the clinical and ethical requirements of their profession. It enables them to perform patient care duties in a range of clinical contexts and situations, to promote and restore the well-being of their patients [31].

#### 3.2.1. Knowledge

The review showed that competence depended on expert nursing knowledge, acquired through education and experience. This was directly referred to in nine out of the 13 studies, from both nurses' and patients' perspectives [15–20, 24, 25, 27]. Two studies [15, 19] both distinguished a professional knowledge base (general nursing knowledge attained through education and experience), a situational knowledge base (patient-specific knowledge acquired through assessment) and critical thinking skills (the ability to evaluate the available evidence before making a judgement) as important components of knowledge. Finally, several studies with patient samples [16–18] emphasised the good nurse developing detailed knowledge of their patients, being aware of their needs and feelings in the moment and being able to identify subtle changes in patients' conditions.

#### 3.2.2. Technical Skill

The requirement for the good nurse to possess expert technical skill and proficiency was identified in eight out of the 13 studies [15–19, 24, 25, 27]. This can be seen as an extension of the requirement for knowledge, in that it directly translates the theoretical understanding of nursing into practical application.

The types of practical skills described in these studies included checking patients' vital signs, administering emergency care and treatment and operating technology and machinery in the hospital. One study [19] also found that being able to multitask was an important related trait, as nurses are often required to perform numerous tasks concurrently to effectively manage the provision of care. In other studies, there were also suggestions that the good nurse should be ‘not squeamish' [17] and should avoid expressing attitudes such as disgust ‘even when performing dirty work' [20], suggesting that technical skill can be affected if nurses become easily upset or disgusted by parts of their work.

#### 3.2.3. Communication

Nine of the 13 studies [15–20, 23, 24, 27] highlighted the need for expert communication skills, as these helped nurses to build relationships with patients and co-workers. The authors of one study [16] concluded that ‘the good nurse was one who could communicate with patients in a way that made them feel at ease and able to openly talk about their concerns, who could talk to patients in ways they understood, and who could maintain good communication among healthcare team members' (p. 22).

Several studies went into more detail about specific skills associated with good communication. For example, several studies [17, 23, 24, 28] described how building rapport depended on listening skills, demonstrating that effective communication is more than just talking. Studies with patients [16, 18] identified other simple but important behaviours the nurse should exhibit to build better relationships, such as introducing themselves to patients and greeting patients when they see them. Finally, three studies [16, 18, 19] highlighted the importance of humour, which was thought to help nurses establish more authentic and positive relationships with their patients.

#### 3.2.4. Professionalism

Professionalism (i.e., the ability to act in accordance with the standards of one's profession) was identified either directly or indirectly in numerous studies. For example, ‘professional comportment' (including the expectation for nurses to have pride in themselves and be responsible) was one of the two main ways in which the good nurse was identified by patients in one study [16]. Similarly, another study [20] found that the good nurse should possess a ‘professional attitude', defined as the capacity to maintain a positive attitude, to act in a trustworthy and respectful way, and to apologise when they made a mistake. This study also noted that the good nurse should present a professional appearance at all times.

Mostly, however, the necessity for professionalism was alluded to more indirectly through descriptions of related qualities. For example, different studies found that the good nurse should be responsible [16, 19, 22], proactive [25] and advocate on behalf of their patients [15, 18, 24]. Moreover, they should be organised [19] and efficient [17, 28] in their work. Finally, it was also found that they should take pride in their work [16, 25] and seek to develop an established nursing identity by having a clear vision and goals, and working towards them with a professional attitude [24].

#### 3.2.5. Supportive

Being supportive (i.e., providing encouragement or emotional help to others) was another interpersonal skill highlighted in the review. For example, five studies [18, 20, 21, 24, 25] concluded that the good nurse should be emotionally supportive and helpful, capable of understanding and addressing the patients' emotional concerns. Interestingly, the study in a mental health hospital [21] found a greater expectation for nurses to provide emotional support than other healthcare workers (doctors, social workers) and that nurses should be more concerned with emotional support than with medication treatment. It is interesting that this was seen as the primary role for the nurse, although it may simply reflect the context of patients with emotional disorders.

The mental health patients also expected nurses to be cooperative by involving them in the treatment process [21]. Two studies with nurses also highlighted the importance of being cooperative or collaborative [25, 27], although here they were referring to providing support to co-workers. Collaboration was a particularly strong theme in one study [25], whose authors described it as ‘an ethical responsibility of nurses because quality care and patient safety are possible in today's complex healthcare environments through interdisciplinary care teams working in collaboration with multiple healthcare providers and through nurses creating a bridge between these care teams and patients' (p. 8). Clearly, it was recognised that encouragement and mutual support helped the nurses to collaborate effectively in the complex healthcare environment.

#### 3.2.6. Holistic Care

Finally, the importance of holistic care (i.e., the ability to address the full range of patient needs, including physical, emotional, social and spiritual well-being, as part of comprehensive care) was identified in three studies [15, 24, 25], all of which were conducted with nurses. Two of these [15, 25] provided limited detail on the meaning of holistic care, other than to imply the general requirement for care which extended beyond patients' physical concerns. The other study [24] provided a description of holistic nursing practices and emphasised the importance of spiritual care, concluding that the good nurse should not only take time to learn about the patient's emotional needs but also provide spiritual support in accordance with the patient's religion.

## 4. Discussion

The scoping review revealed that the good nurse was primarily defined by their character and competence. There was consensus across the studies for these themes and their constituent subthemes. Additionally, the results accord with other, broader studies of good nursing characteristics. For example, three previous literature reviews [7–9] also found that the good nurse could be distinguished by a virtuous character and expert competence, and highlighted a similar list of traits. Our review builds upon these, shows that similar results are found in different samples and provides more detail on the potential nature of cross-cultural and cross-context differences in perceptions.

Our results also accord with established codes of nursing ethics. For example, the ICN Code of Ethics [32] describes the requirement for nurses to commit themselves completely to ethical care (which relies on good character) and to maintain their competence through continual development. Indeed, the subthemes identified in this review align closely with the ICN's Code: for example, character virtues such as ‘respectfulness' and ‘caring' are identified both in this study and as core professional values aligned with the ICN's ‘Nurses and People' ethical provision, whereas competence (including knowledge and skill) is recognised both in this study and as core elements of the ICN's ‘Nurses and Practice' and ‘Nurses and the Profession' provisions. This reveals a general consensus that the virtues and traits identified in this review are integral to the meaning of a good nurse.

The review makes a novel contribution in showing that nurses and patients generally accorded in their understanding of the good nurse. With the exception of the requirement for holistic care (which was only identified by nurses), there was at least one study from the nurses' perspective and one study from the patients' perspective to support each of the subthemes. However, there were also signs of some small differences. Specifically, the patients attributed greater importance to interpersonal behaviours, particularly around how the nurses acted towards their patients (e.g., out of the six studies highlighting ‘respectfulness', only one was conducted with nurses). By contrast, the nurses attributed greater importance to specific competencies (e.g., out of the eight studies highlighting ‘technical skills', only three were conducted with patients). Therefore, while patients and nurses generally shared perceptions of the good nurse, there were also small differences which reflected their experiences (i.e., as the providers or recipients of care).

There was also some evidence that perspectives varied between healthcare contexts. For example, the study in a mental health hospital [21] found that patients expected the good nurse to provide more emotional support and to have a more personal relationship with them, emphasising the virtues of being understanding, encouraging, patient and warm. This emerged more strongly here than it had in other studies, likely reflecting the context of patients who suffered from emotional issues. This indicates that the meaning of the good nurse varies in different areas of practice; however, more cross-context research will be needed to test this more rigorously.

Finally, there was also some evidence that perspectives varied across different cultures, but too few studies to draw a firm conclusion. For example, in accordance with Hofstede's individualism–collectivism dimension [33], the scoping review found that studies in Western cultures (which are characterised as highly individualistic [33]) tended to highlight individual traits (e.g., courage, calmness and efficiency), whereas those in East Asian cultures (characterised by collectivism and relational harmony [34]) emphasised social relationships and the nurse's interpersonal responsibilities. This may also explain why autonomy and advocacy are more prominent in Western nursing discourses (e.g., the American Nurses Association's emphasis on patient advocacy [35]), while values such as *rei* (respect) and *wa* (harmony) are embedded in Japan's professional medical codes [36, 37]. There was also an indication that nurses in Western cultures are expected to be warm and expressive, whereas those in East Asian cultures are expected to practice emotional restraint and professional detachment, reflecting the cultural contrasts expressed in Trompenaars' neutral–affective dimension [38]. These differing cultural patterns underscore the necessity for culturally sensitive nursing education and practice. That said, these differences were relatively minor, and in general, the core themes of character and competence were identified (directly or indirectly) in studies across Europe, North America and Asia.

### 4.1. Implications

The results of the review reinforce the importance of virtue ethics in determining and shaping the research on the good nurse. The studies revealed that the core theme of ‘character' was defined by virtues, which underpinned moral excellence and the development of ethical and professional competence. It can be understood that the good nurse develops virtue by thinking critically about what it means to act ethically in particular situations (particularly those characterised by moral uncertainty), practising and repeating virtuous behaviours in real-life scenarios, and experiencing happiness when this results in a good outcome. It is through this process that the virtues are habituated, and the virtuous character is established [39].

To support this, there may be a role for ethics education in helping nurses to develop virtue and ethical competence. For example, nurses can be directly encouraged to contemplate and practice ethical behaviour—and thereby develop the character of a good nurse—during ethics committees, consultations and ward rounds [40]. Simulated case scenarios (where students must respond to ethically complex patient situations, such as negotiating care decisions with a patient's family or intervening when a colleague dismisses a patient's concern) can offer practical experience in moral decision-making. Similarly, guided reflection journals may help nurses internalise virtues such as optimism and resilience by critically reflecting on their responses to critical challenges. Such activities remain relatively rare in practice; however, more research is needed to identify the most effective content and delivery style for ethics education.

However, a nurse might have a virtuous character but limited skill in nursing, or be clinically competent but lack good character. This underscores the role of deontological (duty-based) ethics in the development of clinical competence and ensuring that high-quality ethical care is realised in practice. Regulatory frameworks for nurse training, licensure and professional conduct—validated through independent assessment—help to ensure that nurses meet established standards of knowledge and skill. Moreover, codified nursing ethics also offer essential guidance on professional duties and obligations [41], particularly in contexts of moral uncertainty, indirectly supporting ethical competence. In this way, the development of ethical competence relies on both a virtuous character and the extrinsic guidance provided through deontological ethics.

Ultimately, virtue ethics and deontological ethics recursively define what it means to be a good nurse. The good nurse is internally motivated to develop a virtuous character, thinking carefully about what it means to act for the good of the patient, and habituating the behaviours that support this. At the same time, deontological ethics guide the nurses' development of competency and help them understand how they should act, clearly defining their duties, expectations and professional ethics. The commitment to develop virtue and follow professional ethics helps the nurse to move ever closer towards the ethical ideal of the good nurse.

While the review centres on hospital-based studies, the themes we have identified may also translate to other care contexts, such as community health, primary care or long-term care. For example, in community settings, where nurses build sustained, one-on-one relationships with patients, character traits such as patience, respectfulness and emotional support may be even more salient. Conversely, certain competencies, such as technical multitasking, may manifest differently or take on less emphasis. Similarly, the rise of telehealth, remote monitoring and artificial intelligence–supported care introduces new forms of interaction that may shift how character and competence are expressed. In tele-nursing, for example, qualities like warmth and attentiveness must be conveyed through digital communication, while competence may require expertise with digital tools and platforms. These emerging models of care raise important questions about how the ideal of the good nurse evolves when face-to-face interaction is reduced or technologically mediated. Future research could explore how perceptions adapt to these varied clinical and technological contexts, and whether distinct context-specific competencies emerge.

### 4.2. Limitations

Certain limitations of the scoping review and of the evidence base on the characteristics of the good nurse should be acknowledged. Firstly, the fact that relatively few studies were identified in the review may have resulted from several forms of bias associated with the inclusion criteria. The review was restricted to studies published in English, introducing the possibility that relevant studies published in other languages were omitted. Secondly, the inclusion criteria limited studies to peer-reviewed academic publications so that methodological quality was preserved; however, this may have contributed to publication bias by excluding relevant grey literature or negative findings. Thirdly, although five major databases were searched, it is possible that relevant studies indexed in other databases or nonindexed sources were missed, creating a risk of selection bias.

This led to a relatively sparse evidence base, meaning certain research questions could not be conclusively answered, such as the extent of cross-cultural and cross-context differences in perceptions of the good nurse. This was exacerbated by the fact that only three [20, 22, 25] of the 13 studies included quantitative assessments of good nursing traits. The largely qualitative approach generated rich narratives into the good nurse and enabled the identification of the core characteristics, but offered limited insight into which traits were most important, and whether identified traits were culture- or context-dependent. To address these limitations, future research could employ more structured, scalable methodologies, such as global Delphi studies, to achieve expert consensus on the relative importance of caring traits. Cross-national surveys could also be used to quantify cultural differences in expectations of the good nurse across different health systems. Additionally, including grey literature (e.g., national nursing board standards, regulatory guidelines) could enrich the evidence base and help to bridge the gap between academic research and professional practice frameworks.

Another limitation is that the identified studies varied significantly in their level of detail. This reflected differences in the studies' scope, methodology and sample size (e.g., the smallest study [23] had a sample size of just three nurses, whereas the largest [20] had a sample size of 557). The differences may also reflect journal word limit restrictions, which also affects the level of detail that can be reported. As a result of these factors, some studies provided comprehensive lists of good nursing traits, whereas others were sparser and made fewer contributions to the overall pool of good nursing traits. It is unclear whether the more detailed descriptors were absent in the data of these sparser studies, or if they did emerge but were combined with related terms for brevity.

Similarly, none of the included studies disaggregated their findings based on participant characteristics such as age, gender or ethnicity. This represents a critical gap, as individual identity and lived experience are likely to shape how nurses and patients perceive the qualities of the good nurse. Future research should explore these intersections to better understand how personal and sociocultural factors may influence expectations and experiences of nursing care.

Finally, as with any thematic synthesis, the interpretation of findings involved a degree of subjectivity, shaped by the backgrounds and beliefs of the research team. We sought to mitigate potential bias through collaborative coding discussions and iterative theme refinement, while remaining mindful of our interpretive role throughout the analysis. In future research, it may be beneficial to build on this by incorporating structured reflexivity practices (e.g., memos, positionality statements) to further enhance transparency in the analytic process.

## 5. Conclusion

The review led to the identification of the central themes of character and competence. With respect to character, the good nurse was perceived as patient, caring, optimistic, trustworthy, selfless, respectful and confident. With respect to competence, the good nurse was perceived as knowledgeable, technically skilled, professional, an expert communicator, supportive to patients and colleagues, and capable of providing holistic care. By developing a virtuous character and expert competence, it was thought that the good nurse would maintain more positive interpersonal relationships, respond better to challenges and—ultimately—provide a higher quality of care to patients.

## Figures and Tables

**Table 1:** Summary of the search strategy.

PCC elements	Search terms—characteristics of the good nurse
*Population* Nurses and patients	MH “nurses+” OR MH “Nurse Clinicians” OR MH “nursing+” OR MH “Nursing staff+” OR TI “nurs^∗^” MH “patients+” OR TI “patient^∗^” OR MH “Patient care+”

*Concept* Perception of the good nurse	MH “perception+” OR MH “attitude+” OR TI “View^∗^” OR TI “Opinion^∗^” OR TI “Reflect^∗^” OR TI “Perspective^∗^” OR TI “Perception^∗^” OR TI “Expectation^∗^” OR TI “Experience^∗^” OR TI “description^∗^” MH “Virtue” OR MH “ethics+” OR MH “Ethics, Nursing” OR TI “Excellence” OR TI “Qualit^∗^” OR TI “Trait^∗^” OR TI “Character^∗^” OR TI “Good Nurs^∗^” OR TI “Virtue^∗^” OR TI “Value^∗^”

*Context* Hospital setting	MH “hospitals+” OR MH “hospitalization+” OR MH “Secondary Care” OR MH “Intensive Care units+” OR MH “Critical care+” OR MH “Critical Care Nursing” OR MH “Palliative Care” OR TI “ICU” OR TI “intensive care” OR TI “Critical Care” OR TI “Secondary Care” OR TI “Hospital^∗^”

*Combination*	Nurses and patients AND Perception of the good nurse AND Hospital setting

*Limiters*	Academic journals Peer-reviewed English language Adult participants

**Table 2:** Details of the identified articles into the characteristics of the good nurse.

Reference	Year	Title	Aims and objectives	Country	Sample	Research method	Quality assessment	Key findings
Nadelson [13]	2002	Being a good nurse and doing the right thing: A qualitative study	To examine nurses' perceptions of what it means to be a good nurse and to do the right thing	USA	53 nurses from various areas of practice	Written responses to two open-ended questions *(qualitative content analysis)*	High	Seven categories described the good nurse: personal characteristics (caring, compassion, respect, communication), professional characteristics (commitment), knowledge base (professional knowledge, situational knowledge, technical skill), patient-centredness (selflessness), advocacy (intervening on behalf of patients), critical thinking (judgement, reflective analysis) and patient care (performance of care).

Izumi et al. [14]	2006	Japanese patients' descriptions of ‘the good nurse': Personal involvement and professionalism	To explore the characteristics of ‘the good nurse' as an ethical ideal from patients' perspectives	Japan	26 cancer patients	Semistructured interviews (*phenomenological analysis*)	High	Good nurses were identified by their involvement as a person, including being a good person (cheerful, kind, considerate, sympathetic, compassionate, sensitive, hopeful, humorous, courteous), presenting themselves as persons (introducing themselves, casual conversations), being interested in patients as persons (getting to know patients and families) and caring for their patient as a precious person (caring, empathetic, respectful). Good nurses were also identified by their professional comportment, including professional competency (technical skill, knowledgeable, communication, awareness) and professional disposition (pride, responsibility, striving to improve).

Smith and Godfrey [15]	2006	What makes a good nurse? views of patients and carers	To identify what makes a good nurse, focusing on which attitudes, skills and knowledge student nurses should be learning	UK	96 patients (adults with learning disabilities, children aged 10–11 years), service users and carers	Focus groups (*thematic analysis*)	Moderate	The strongest theme for the good nurse was communication, particularly using listening skills and being willing to communicate. Attitude, particularly respectfulness and a caring nature, was also highlighted. Finally, other important traits included confidence, being approachable, nonsqueamish, efficient, honest, patient, calm, clever, firm, knowledgeable, having clinical skills, sympathetic, well-trained, appropriate appearance, good hygiene and having common sense.

Izumi et al. [16]	2009	Exploring the views of patients with cancer on what makes a good nurse—a pilot study	To explore the views of cancer patients on what makes a good nurse	UK	5 cancer patients	Semistructured interviews (*grounded theory*)	Moderate	The analysis highlighted good nurse virtues (helpfulness, courage, respectfulness, reliability, care, sense of humour, kindness, fidelity, friendliness, empathy, patience, sensitive, understanding, honest), knowledge (professional competence, knowledge of the patient), skills (technical skill, skill in greeting, skill in advocacy and communication) and organisational culture (good organisational climate).

Rush and Cook [17]	2011	Being a good nurse and doing the right thing: A replication study	To replicate the study by Smith & Godfrey (2002) and identify what it means to be a good nurse	USA	20 hospital nurses from various areas of practice	Structured interviews *(relational analysis)*	Moderate	Good nurses were defined by four main categories: personal traits and attributes (caring, patient, selfless, responsible), technical skills and management of care (intelligence, critical thinking, multitasking), work environment and co-workers (cheerful, committed, accountable) and caring and caring behaviours (kindness, communication, caring attitude).

Gallagher et al. [18]	2013	Oncology patients' perceptions of ‘‘the good nurse”: A descriptive study in Flanders, Belgium	To explore how patients perceive ‘the good nurse', and whether perceptions differ due to patient-related or context-related variables	Belgium	557 cancer patients	The *Care-Q* survey instrument of nurse caring behaviours *(exploratory factor analysis)*	Moderate	Three main factors described the good nurse: a supportive and communicative attitude towards patient and family (communication, support, caring), competence and professional attitude (knowledgeable, patient, calm, respectful) and personal involvement towards patient and family (selfless, professional appearance, attentive).

Catlett and Lovan [19]	2014	Patients' perceptions of what makes a good doctor and nurse in an Israeli mental health hospital	To identify inpatients' expectations of their doctors and nurses in a mental health hospital setting	Israel	72 inpatients at a mental health hospital	Structured interviews *(descriptive statistical analysis and content analysis)*	High	The most desired nursing traits and behaviours included being encouraging, compassionate, available/visible, understanding, cooperative, supportive, patient, warm and respectful.

Van der Elst et al. [20]	2015	Nurses' reflections on good nurse traits: Implications for improving care quality	To elicit nurses' self-exploration of the importance of good nurse traits, and to explore discrepancies between traits perceived as ideally and actually important	Taiwan	472 patients with chronic diseases *(ranked good nursing traits)* 35 nurses from a teaching hospital *(completed card-sorting and interviews)*	1. Card-sorting and prioritising exercise 2. Structured interviews *(content analysis)*	High	The good nursing traits identified as most important were being patient, responsible, cautious, friendly, considerate, enthusiastic, caring, sincere, attentive and diligent. The most over-performed traits (showing the difference between perceived importance and how nurses actually acted) were being obedient, lovely and cheerful, whereas the most under-performed traits were being enterprising, cautious and considerate.

Haron and Tran [21]	2017	Using narratives to enhance nursing practice and leadership: What makes a good nurse?	To explore the various roles nurses play and what makes a good nurse	USA	3 registered nurses	‘Storytelling' narratives *(analysis method not stated)*	Moderate	According to the narratives, the good nurse should allow the family to ‘interfere' in patient care (involves family in care), should value their patients (communication, good listener, attentive, detail-oriented) and should focus on the patient (selfless, develops knowledge of patient).

Chen and Hsu [22]	2019	Revisiting the meaning of a good nurse	To explore the meaning of a good nurse from the perspective of nurses	South Korea	10 nurses working in general hospitals or health-related national organisations	Semistructured interviews *(content analysis)*	Moderate	Six main categories defined the good nurse: leans in towards the patient (warm emotions and love, good rapport, advocates for patient), provides professional care (provides information, evidence-based care, prompt and skilful care, holistic care and socio-environmental support), keeps the cornerstone of one's mind with a positive attitude (self-managed, positivity), maintains a good relationship with colleagues (positive partnerships, comradeship), matures with his/her nursing philosophy and conviction (establishes identity as a nurse, has own nursing philosophy) and contributes to society in various ways.

Wadsworth et al. [23]	2020	Questionnaire development of a good nurse and better nursing from Korean nurses' perspective	To develop and assess the psychometric properties of two questionnaires used to assess the ‘good nurse' and ‘better nursing'	South Korea	30 registered nurses *(interviewed to elicit good nursing traits)* 10 subject-matter experts (5 nursing professors, 5 experienced clinical nurses) *(evaluated content validity of questionnaire items)* 1046 registered nurses *(completed questionnaires during pilot and main study)*	1. Re-analysis of semistructured interviews *(content analysis)* 2. Evaluation of content validity by subject-matter experts 3. Questionnaire *(confirmatory factor analysis)*	Moderate	In the developed model, five main dimensions characterised the good nurse: collaboration (good relationships), professional competency (professional knowledge, skills), self-efficacy (self-belief, positivity), a sense of achievement (pride), and compassion (empathy, supportive). Better nursing was associated with person-centred nursing (holistic care, preserving personhood), proactive nursing (carrying out proactive roles), and expertise in nursing (fluid and seamless care).

Kim et al. [24]	2020	Becoming a good nurse – Socialisation of newly employed nurses into the oncological clinic	To explore newly employed nurses' socialisation into an ontological clinic	Sweden	9 healthcare workers (7 nurses, 2 unit managers) at an oncological clinic	Semistructured interviews *(latent thematic analysis)*	High	In describing how the good nurse can be socialised, the qualities of being knowledgeable, having high-level professional skills, and being compassionate were highlighted. Additionally, communication and collaboration were highlighted as valuable skills for nurses to develop.

Park et al. [25]	2020	Exploration of service recipients' image of a perfect nurse: A qualitative descriptive study	To explore the image of the perfect nurse from service recipients' perspective	Iran	12 adults with recent experience using nursing services	Semistructured interviews *(qualitative content analysis)*	Moderate	The perfect nurse was revealed to be affable (compassionate, sympathetic, good-tempered), responsive (answers questions and requests), a ‘tower of strength' (good listener, patient, understanding, attentive) and efficient (provides care which is timely, proper and careful).

**Table 3:** A summary of the themes, subthemes and related terms identified in the scoping review.

Subtheme	Related terms
*Theme 1: character*	
Patience	Calm, accepts change
Caring	Empathetic, kind, compassionate, considerate, gentle, sympathetic, attentive, diligent, committed, sensitive
Optimism	Positive, friendly, cheerful, warm, hopeful, enthusiastic, encouraging
Trustworthiness	Honest, loyal, dependable, ethical, nonjudgemental, approachable, sincere
Selflessness	Patient-centred, unselfish
Respectfulness	Courteous, tolerant, preserves personhood, advocates for patients, involves family in care
Confidence	Courageous, firmness, self-belief, pride, established nursing identity

*Theme 2: competence*	
Knowledge	Critical thinker, problem-solving skills, detail-oriented, aware, develops knowledge of patients, strives to improve, has common sense
Technical skill	Professional competence, multitasker, not squeamish
Professionalism	Organised, responsible, accountable, proactive, responsive, available, cautious, self-motivated, efficient
Communication	Listening skills, sense of humour, understanding, introduces themselves to patients, builds rapport, anticipates needs
Supportive	Emotional support, cooperative, collaboration, helpful, reliable, delegation skills
Holistic care	Provides holistic care, provides spiritual care

## Data Availability

The data supporting the findings of this scoping review consist of published studies identified through a structured literature search. These studies are cited in the manuscript and can be accessed through their respective publishers. The ‘Example Search Results' document has been provided as Supporting Information for transparency. The complete EndNote library used for this review cannot be shared publicly due to copyright restrictions. However, interested researchers may contact the corresponding author for further details on the search strategy and data extraction process. Permission is provided for any new publications based on such secondary analyses.

## Supporting Information

Example Search Results from Medline and Scopus.
