# Supplementary material for: The Characteristics of the ‘Good Nurse': A Scoping Literature Review
Source: Nurs Res Pract. 2025 Aug 5;2025:8460996. doi: 10.1155/nrp/8460996 (PMC12343166; doi:10.1155/nrp/8460996)
Supplement: Supporting Information — Additional supporting information can be found online in the Supporting Information section. [file 8460996.f1.docx]

**Medline**

Tuesday, December 17, 2019 6:43:47 AM

| **#** | **Query** | **Limiters/Expanders** | **Results** |
| --- | --- | --- | --- |
| S48 | S9 AND S20 AND S31 AND S44 | Limiters - Scholarly (Peer Reviewed) Journals Expanders - Apply equivalent subjects Narrow by SubjectAge: - all adult: 19+ years Narrow by Language: - english Search modes - Boolean/Phrase | 877 |
| S47 | S9 AND S20 AND S31 AND S44 | Limiters - Scholarly (Peer Reviewed) Journals Expanders - Apply equivalent subjects Narrow by Language: - english Search modes - Boolean/Phrase | 1,511 |
| S46 | S9 AND S20 AND S31 AND S44 | Limiters - Scholarly (Peer Reviewed) Journals Expanders - Apply equivalent subjects Search modes - Boolean/Phrase | 1,617 |
| S45 | S9 AND S20 AND S31 AND S44 | Expanders - Apply equivalent subjects Search modes - Boolean/Phrase | 5,914 |
| S44 | S32 OR S33 OR S34 OR S35 OR S36 OR S37 OR S38 OR S39 OR S40 OR S41 OR S42 OR S43 | Expanders - Apply equivalent subjects Search modes - Boolean/Phrase | 768,945 |
| S43 | TI Hospital* | Expanders - Apply equivalent subjects Search modes - Boolean/Phrase | 306,965 |
| S42 | TI Secondary Care | Expanders - Apply equivalent subjects Search modes - Boolean/Phrase | 1,525 |
| S41 | TI Critical Care | Expanders - Apply equivalent subjects Search modes - Boolean/Phrase | 11,828 |
| S40 | TI intensive care | Expanders - Apply equivalent subjects Search modes - Boolean/Phrase | 40,346 |
| S39 | TI ICU | Expanders - Apply equivalent subjects Search modes - Boolean/Phrase | 8,758 |
| S38 | (MH "Palliative Care") | Expanders - Apply equivalent subjects Search modes - Boolean/Phrase | 52,298 |
| S37 | (MH "Critical Care Nursing") | Expanders - Apply equivalent subjects Search modes - Boolean/Phrase | 1,849 |
| S36 | (MH "Critical Care+") | Expanders - Apply equivalent subjects Search modes - Boolean/Phrase | 55,882 |
| S35 | (MH "Intensive Care Units+") | Expanders - Apply equivalent subjects Search modes - Boolean/Phrase | 80,468 |
| S34 | (MH "Secondary Care") | Expanders - Apply equivalent subjects Search modes - Boolean/Phrase | 547 |
| S33 | (MH "Hospitalization+") | Expanders - Apply equivalent subjects Search modes - Boolean/Phrase | 229,218 |
| S32 | (MH "Hospitals+") | Expanders - Apply equivalent subjects Search modes - Boolean/Phrase | 267,613 |
| S31 | S21 OR S22 OR S23 OR S24 OR S25 OR S26 OR S27 OR S28 OR S29 OR S30 | Expanders - Apply equivalent subjects Search modes - Boolean/Phrase | 1,105,790 |
| S30 | TI virtue* | Expanders - Apply equivalent subjects Search modes - Boolean/Phrase | 1,004 |
| S29 | TI Excellence | Expanders - Apply equivalent subjects Search modes - Boolean/Phrase | 4,359 |
| S28 | TI qualit* | Expanders - Apply equivalent subjects Search modes - Boolean/Phrase | 255,121 |
| S27 | TI trait* | Expanders - Apply equivalent subjects Search modes - Boolean/Phrase | 36,785 |
| S26 | TI character* | Expanders - Apply equivalent subjects Search modes - Boolean/Phrase | 520,944 |
| S25 | TI value* | Expanders - Apply equivalent subjects Search modes - Boolean/Phrase | 156,683 |
| S24 | TI good nurs* | Expanders - Apply equivalent subjects Search modes - Boolean/Phrase | 623 |
| S23 | (MH "Ethics, Nursing") | Expanders - Apply equivalent subjects Search modes - Boolean/Phrase | 10,214 |
| S22 | (MH "Ethics+") | Expanders - Apply equivalent subjects Search modes - Boolean/Phrase | 143,713 |
| S21 | (MH "Virtues") | Expanders - Apply equivalent subjects Search modes - Boolean/Phrase | 1,166 |
| S20 | S10 OR S11 OR S12 OR S13 OR S14 OR S15 OR S16 OR S17 OR S18 OR S19 | Expanders - Apply equivalent subjects Search modes - Boolean/Phrase | 1,451,945 |
| S19 | TI reflect* | Expanders - Apply equivalent subjects Search modes - Boolean/Phrase | 48,619 |
| S18 | TI description* | Expanders - Apply equivalent subjects Search modes - Boolean/Phrase | 33,966 |
| S17 | TI expectation* | Expanders - Apply equivalent subjects Search modes - Boolean/Phrase | 10,126 |
| S16 | TI experience* | Expanders - Apply equivalent subjects Search modes - Boolean/Phrase | 242,572 |
| S15 | TI perception* | Expanders - Apply equivalent subjects Search modes - Boolean/Phrase | 71,923 |
| S14 | TI perspective* | Expanders - Apply equivalent subjects Search modes - Boolean/Phrase | 118,476 |
| S13 | TI opinion* | Expanders - Apply equivalent subjects Search modes - Boolean/Phrase | 14,875 |
| S12 | TI view* | Expanders - Apply equivalent subjects Search modes - Boolean/Phrase | 73,668 |
| S11 | (MH "Attitude+") | Expanders - Apply equivalent subjects Search modes - Boolean/Phrase | 554,854 |
| S10 | (MH "Perception+") | Expanders - Apply equivalent subjects Search modes - Boolean/Phrase | 419,507 |
| S9 | S1 OR S2 OR S3 OR S4 OR S5 OR S6 OR S7 OR S8 | Expanders - Apply equivalent subjects Search modes - Boolean/Phrase | 2,933,132 |
| S8 | TI Patient* | Expanders - Apply equivalent subjects Search modes - Boolean/Phrase | 1,786,527 |
| S7 | (MH "Patient Care+") | Expanders - Apply equivalent subjects Search modes - Boolean/Phrase | 934,955 |
| S6 | (MH "Patients+") | Expanders - Apply equivalent subjects Search modes - Boolean/Phrase | 62,383 |
| S5 | TI nurs* | Expanders - Apply equivalent subjects Search modes - Boolean/Phrase | 253,817 |
| S4 | (MH "Nursing Staff+") | Expanders - Apply equivalent subjects Search modes - Boolean/Phrase | 65,076 |
| S3 | (MH "Nursing+") | Expanders - Apply equivalent subjects Search modes - Boolean/Phrase | 249,732 |
| S2 | (MH "Nurse Clinicians") | Expanders - Apply equivalent subjects Search modes - Boolean/Phrase | 8,148 |
| S1 | (MH "Nurses+") | Expanders - Apply equivalent subjects Search modes - Boolean/Phrase | 86,437 |

**Scopus**

Thursday, March 10, 2022

| **#** | **Query** | **Limiters/Expanders** | **Results** |
| --- | --- | --- | --- |
| S48 | S9 AND S20 AND S31 AND S44 | Limiters - Scholarly (Peer Reviewed) Journals Narrow by SubjectAge: - all adult: 19+ years Narrow by Language: - english Search modes - Boolean/Phrase | 645 |
| S47 | S9 AND S20 AND S31 AND S44 | Limiters - Scholarly (Peer Reviewed) Journals Narrow by Language: - english Search modes - Boolean/Phrase | 902 |
| S46 | S9 AND S20 AND S31 AND S44 | Limiters - Scholarly (Peer Reviewed) Journals Search modes - Boolean/Phrase | 1,032 |
| S45 | S9 AND S20 AND S31 AND S44 | Search modes - Boolean/Phrase | 2,669 |
| S44 | S32 OR S33 OR S34 OR S35 OR S36 OR S37 OR S38 OR S39 OR S40 OR S41 OR S42 OR S43 | Search modes - Boolean/Phrase | 72,089 |
| S43 | TI Hospital* | Search modes - Boolean/Phrase | 41,411 |
| S42 | TI Secondary Care | Search modes - Boolean/Phrase | 1,525 |
| S41 | TI Critical Care | Search modes - Boolean/Phrase | 13,231 |
| S40 | TI intensive care | Search modes - Boolean/Phrase | 4,229 |
| S39 | TI ICU | Search modes - Boolean/Phrase | 857 |
| S38 | (MH "Palliative Care") | Search modes - Boolean/Phrase | 650 |
| S37 | (MH "Critical Care Nursing") | Search modes - Boolean/Phrase | 187 |
| S36 | (MH "Critical Care+") | Search modes - Boolean/Phrase | 2,921 |
| S35 | (MH "Intensive Care Units+") | Search modes - Boolean/Phrase | 1,255 |
| S34 | (MH "Secondary Care") | Search modes - Boolean/Phrase | 225 |
| S33 | (MH "Hospitalization+") | Search modes - Boolean/Phrase | 2,099 |
| S32 | (MH "Hospitals+") | Search modes - Boolean/Phrase | 20,132 |
| S31 | S21 OR S22 OR S23 OR S24 OR S25 OR S26 OR S27 OR S28 OR S29 OR S30 | Search modes - Boolean/Phrase | 801,024 |
| S30 | TI virtue* | Search modes - Boolean/Phrase | 238 |
| S29 | TI Excellence | Search modes - Boolean/Phrase | 64 |
| S28 | TI qualit* | Search modes - Boolean/Phrase | 16,522 |
| S27 | TI trait* | Search modes - Boolean/Phrase | 499 |
| S26 | TI character* | Search modes - Boolean/Phrase | 78,342 |
| S25 | TI value* | Search modes - Boolean/Phrase | 42,830 |
| S24 | TI good nurs* | Search modes - Boolean/Phrase | 74 |
| S23 | (MH "Ethics, Nursing") | Search modes - Boolean/Phrase | 1 |
| S22 | (MH "Ethics+") | Search modes - Boolean/Phrase | 61 |
| S21 | (MH "Virtues") | Search modes - Boolean/Phrase | 9 |
| S20 | S10 OR S11 OR S12 OR S13 OR S14 OR S15 OR S16 OR S17 OR S18 OR S19 | Search modes - Boolean/Phrase | 448,777 |
| S19 | TI reflect* | Search modes - Boolean/Phrase | 12,073 |
| S18 | TI description* | Search modes - Boolean/Phrase | 2,358 |
| S17 | TI expectation* | Search modes - Boolean/Phrase | 494 |
| S16 | TI experience* | Search modes - Boolean/Phrase | 4,605 |
| S15 | TI perception* | Search modes - Boolean/Phrase | 1,170 |
| S14 | TI perspective* | Search modes - Boolean/Phrase | 2,214 |
| S13 | TI opinion* | Search modes - Boolean/Phrase | 322 |
| S12 | TI view* | Search modes - Boolean/Phrase | 4,533 |
| S11 | (MH "Attitude+") | Search modes - Boolean/Phrase | 253 |
| S10 | (MH "Perception+") | Search modes - Boolean/Phrase | 252 |
| S9 | S1 OR S2 OR S3 OR S4 OR S5 OR S6 OR S7 OR S8 | Search modes - Boolean/Phrase | 62,966 |
| S8 | TI Patient* | Search modes - Boolean/Phrase | 9,072 |
| S7 | (MH "Patient Care+") | Search modes - Boolean/Phrase | 139 |
| S6 | (MH "Patients+") | Search modes - Boolean/Phrase | 4,331 |
| S5 | TI nurs* | Search modes - Boolean/Phrase | 553 |
| S4 | (MH "Nursing Staff+") | Search modes - Boolean/Phrase | 19 |
| S3 | (MH "Nursing+") | Search modes - Boolean/Phrase | 149 |
| S2 | (MH "Nurse Clinicians") | Search modes - Boolean/Phrase | 0 |
| S1 | (MH "Nurses+") | Search modes - Boolean/Phrase | 138 |
